# *Ginkgo biloba* Responds to Herbivory by Activating Early Signaling and Direct Defenses

**DOI:** 10.1371/journal.pone.0032822

**Published:** 2012-03-20

**Authors:** Tapan Kumar Mohanta, Andrea Occhipinti, Simon Atsbaha Zebelo, Maria Foti, Judith Fliegmann, Simone Bossi, Massimo E. Maffei, Cinzia M. Bertea

**Affiliations:** 1 Plant Physiology Unit, Department of Life Sciences and Systems Biology, Innovation Centre, University of Turin, Turin, Italy; 2 INRA, Laboratoire des Interactions Plantes-Microorganismes (LIPM), UMR441, Castanet-Tolosan, France; Max Planck Institute for Chemical Ecology, Germany

## Abstract

**Background:**

*Ginkgo biloba* (Ginkgoaceae) is one of the most ancient living seed plants and is regarded as a living fossil. *G. biloba* has a broad spectrum of resistance or tolerance to many pathogens and herbivores because of the presence of toxic leaf compounds. Little is known about early and late events occurring in *G. biloba* upon herbivory. The aim of this study was to assess whether herbivory by the generalist *Spodoptera littoralis* was able to induce early signaling and direct defense in *G. biloba* by evaluating early and late responses.

**Methodology/Principal Findings:**

Early and late responses in mechanically wounded leaves and in leaves damaged by *S. littoralis* included plasma transmembrane potential (Vm) variations, time-course changes in both cytosolic calcium concentration ([Ca^2+^]_cyt_) and H_2_O_2_ production, the regulation of genes correlated to terpenoid and flavonoid biosynthesis, the induction of direct defense compounds, and the release of volatile organic compounds (VOCs). The results show that *G. biloba* responded to hebivory with a significant Vm depolarization which was associated to significant increases in both [Ca^2+^]_cyt_ and H_2_O_2_. Several defense genes were regulated by herbivory, including those coding for ROS scavenging enzymes and the synthesis of terpenoids and flavonoids. Metabolomic analyses revealed the herbivore-induced production of several flavonoids and VOCs. Surprisingly, no significant induction by herbivory was found for two of the most characteristic *G. biloba* classes of bioactive compounds; ginkgolides and bilobalides.

**Conclusions/Significance:**

By studying early and late responses of *G. biloba* to herbivory, we provided the first evidence that this “living fossil” plant responds to herbivory with the same defense mechanisms adopted by the most recent angiosperms.

## Introduction

Dating back more than 200 million years (Myr), *Ginkgo biloba*, the only species remaining from the family Ginkgoaceae, is one of the oldest seed plants often referred to as a “living fossil” because it is known to have existed early in the Jurassic period [1]. Evolutionary studies on fossil leaves and reproductive organs revealed that the morphology of *G. biloba* has little changed during the last 100 Myr [2], [3], and molecular analysis of the *G. biloba* genome (incomplete) suggests a much closer relationship to cycads than to conifers [4], [5]. Paleoecological inferences based on both morphology and sedimentary environments support the idea that *G. biloba* was displaced in riparian habitats by angiosperms with better adaptations to frequent disturbance [3]. *G. biloba* cDNA libraries have been constructed [6], [7] and, recently, a total of 64,057 ESTs were generated using the 454 GS FLX sequencing platform and integrated with the Ginkgo ESTs in GenBank [8].

*G. biloba* has a broad spectrum of resistance or tolerance to many pathogens and herbivores and because of its hardiness the trees are frequently planted in large cities [1]. *G. biloba* anatomy, structure and growth of the shoot apex, heterophylly, patterns of venation and internal secretory structures have been described since the beginning of the last century [9], [10].

Upon herbivore attack, chemical defense mechanisms are usually divided into constitutive and induced, both of them acting either directly or indirectly. Inducibility, or the ability to increase defensive traits after herbivore attack, is viewed as a way for plants to cope with high resource demands and the unpredictability of herbivore attack [11]. All induced defenses require a cascade of events starting from the recognition of the initial herbivore attack to the production of specific defense molecules, upon gene expression and metabolic activation [12]–[14]. With regards direct defenses, some plants that store monoterpenes, like *Mentha aquatica*, respond to herbivory by increasing terpenoid production and up-regulating the expression of genes involved in terpenoid biosynthesis [15]. Species of milkweed (*Asclepias* spp.) use cardenolides to fight both above and belowground herbivores [16], whereas cotton (*Gossypium* spp.) produces gossypol and a variety of other gossypol-like terpenoids that exhibit toxicity to a wide range of herbivores [17]. Important constituents present in *G. biloba* leaves are terpene trilactones (e.g., ginkgolides A, B and C), many flavonol glycosides, biflavones, proanthocyanidins, alkylphenols, simple phenolic acids, 6-hydroxykynurenic acid, 4-O-methylpyridoxine, polyprenols, bilobalide, and ginkgotoxin [18], [19]. Ginkgolide biosynthesis is initiated by the cyclization of the diterpene levopimaradiene and the isolation and characterization of a cDNA encoding *G. biloba* levopimaradiene synthase has been described [20]. The antioxidant, antiischemic, cardioprotective, neurosensory, cerebral, pharmacokinetics, and antiaging activity has been established on standardized *G. biloba* extract, EGb 761 [21]–[25]. EGb 761 has also been demonstrated to be a potent scavenger of free radicals [26], [27]. Thus, EGb might have a potential for scavenging reactive oxygen species (ROS) [28]. The antioxidant properties of *G. biloba* flavonoids can also result from their ability to complex metal ions such as Cu^2+^, Fe^2+^, Zn^2+^ and Mg^2+^ [29].

The pharmacological properties of *G. biloba* correlate with its strong repellent effect on herbivores. In fact, the fecundity of spider mites was almost zero, because they did not survive the intake of toxic *G. biloba* leaf constituents, making impossible rearing spider mites on *G. biloba*, while the rearing of spider mites on other plants was successful [30], [31]. The potential of *G. biloba* and its synthetic metabolites for preventing apple feeding and infestation by neonate lame of the codling moth, *Cydia pomonella*, has been demonstrated [32].

In order to react promptly to herbivore attacks, plants must be able to detect their predators and to react quickly with early signaling. Early events include calcium signaling and the production of ROS, leading to unbalances in the ion distribution across the plasma membrane that eventually alter the plasma transmembrane (Vm) potential, as recently reviewed [14]. These early events and the effect of herbivore-associated elicitors [33] are followed by activation of protein kinase cascades [34], eventually leading to gene expression and production of direct and indirect defenses [35], [36]. Plant tissues that are attacked by herbivores also emit volatile organic compounds (VOCs), that may both induce defense on the same or different plants and attract predators of the attacking herbivore [11], [37], [38].

To better understand the role of direct and indirect defenses in *G. biloba*, we evaluated early and late responses in leaves either mechanically wounded or damaged by the generalist herbivore *Spodoptera littoralis*. Here we show that *S. littoralis* feeding on *G. biloba* induces the typical signaling pathways found in angiosperms. These responses include Vm variations, time-course changes in both cytosolic calcium concentration ([Ca^2+^]_cyt_) and H_2_O_2_ production, the regulation of gene expression, the induction of direct defense compounds and the release of VOCs.

## Results

### Herbivory induces early response defense signaling in *G. biloba*: Vm, Ca^2+^ and H_2_O_2_ variations

**Figure 1:**
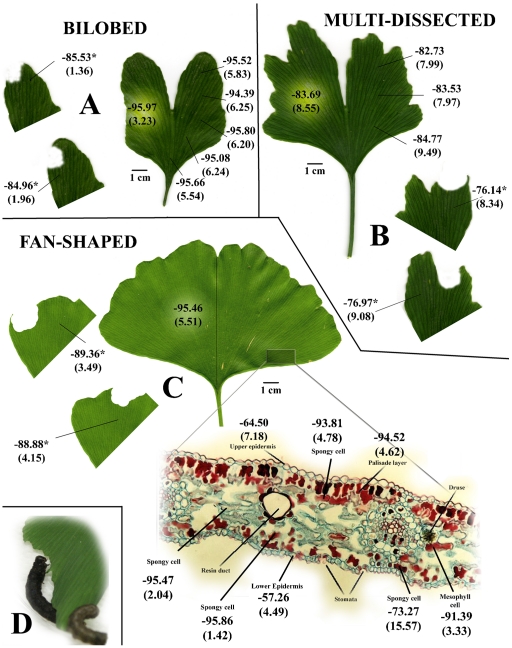
*G. biloba* is characterized by different leaf types, depending on the age and shape of the leaf. **A**, bilobed; **B**, multi-dissected and; **C**, fan-shaped. Vm values are reported along with standard errors (in brackets) as mV (n≈50). Herbivore wounding is shown in leaf segments and Vm values are indicated below and aside the wounding zone. The leaf section of C shows Vm values of the different mesophyll and epidermal cells of a fan-shaped leaf. **D**, *Spodoptera littoralis* feeding on *G. biloba* leaves.

*G. biloba* is characterized by different leaf types, depending on the age and shape of the leaf: bilobed, multi-dissected and fan-shaped. Before herbivore wounding (HW), we mapped the distribution of Vm values in healthy leaves belonging to the three leaf types. We found that bilobed and fan-shaped leaves had almost the same average values (P>0.05), whereas multi-dissected leaves showed statistically lower values (P<0.05). A more careful analysis of fan-shaped leaves showed that epidermal cells had statistically lower Vm values (P<0.05) when compared to palisade and spongy parenchyma cells (Fig. 1).

When Vm was evaluated after mechanical damage (MD) a small and not significant depolarization was observed, no matter the time lapsing after the MD event. On the other hand, a significant Vm depolarization was found up to 6 h after HW (Fig. 1) in all three leaf types (bilobed: 11.01±0.98 mV, P<0.05; multi-dissected: 7.55±0.71 mV, P<0.01; fan-shaped: 6.58±0.53 mV, P<0.01).

*G. biloba* has a particular venation pattern. Leaf blades show unconnected veins, veins which are anastomosed marginally but unconnected basally, and veins which end a considerable distance from the margin. It was speculated that the anastomoses found in *G. biloba* are of a simple, archaic type and are apparently analogous to the anastomoses in the leaves of certain ferns and in the leaflets of various cycads [39]. When Vm was measured below and aside the wounding zone no significant differences were found, indicating that the depolarizing signals is transmitted independent of the anastomotic pattern (Fig. 1). Based on the above results, we chose to run all following analyses on fan-shaped leaves.

In order to evaluate whether *G. biloba* uses the same signaling pathway demonstrated in angiosperms (e.g., Lima bean [40]), MD and HW fan-shaped leaves were pre-incubated with the dyes calcium orange (for the quantitative determination of cytosolic calcium concentration, [Ca^2+^]_cyt_) and Amplex Red (for the quantitative determination of H_2_O_2_ production) [41], [42].

**Figure 2:**
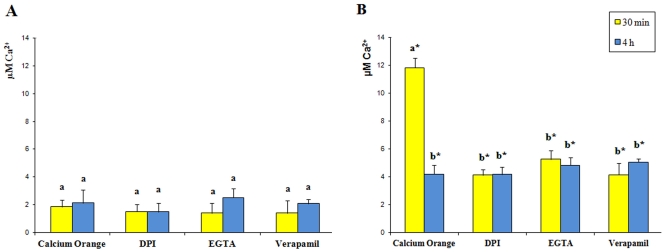
Calcium variations in *G. biloba* upon mechanical damage and herbivore wounding. **A**. Mechanically wounded *G. biloba* leaves, values (n = 5) are expressed as µM Ca^2+^ calculated from a calibration curve. The same letter indicates not significant (P>0.05) variation. B. Herbivore wounded *G. biloba* leaves, values (n = 5) are expressed as µM Ca^2+^. Different letters indicate significant (P<0.05) differences, the asterisks indicate significant (P<0.05) differences with respect to mechanical damage. In both panels, calcium orange indicates the absence of pharmacological inhibitors.

Figure 2 shows time-course variations of [Ca^2+^]_cyt_ following MD (Fig. 2A) and HW (Fig. 2B), with respect to intact leaves. No significant differences were observed between MD and intact leaves (data not shown). After 30 min, a significant increase in [Ca^2+^]_cyt_ was only found following HW (Fig. 2B). However, after 4 h of HW, [Ca^2+^]_cyt_ drastically decreased (Fig. 2B). DPI (diphenyleneiodonium) is a suicide inhibitor of the phagocytic NADPH oxidase and an inhibitor of NADH-dependent H_2_O_2_ production by peroxidase [43]. DPI prompted a strong inhibition of the increase of [Ca^2+^]_cyt_ in HW at 30 min; however, values were significantly higher with respect to MD (compare Figs. 2A and 2B). The calcium ion chelating agent, EGTA has been used to demonstrate the specificity of the effect of Ca^2+^ [44]. When EGTA was used after 30 min of feeding, the chelating agent was found to inhibit the increase of [Ca^2+^]_cyt_ (Fig. 2B). Even in this case, HW showed significantly (P<0.05) higher [Ca^2+^]_cyt_ values than MD in response to EGTA (Fig. 2B). Verapamil is a voltage-gated Ca^2+^ channel antagonist which has a significant effect on herbivore-induced Ca^+2^ release [45], [46]. Verapamil significantly reduced HW [Ca^2+^]_cyt_ after 30 min, although values were still higher with respect to MD (Figs. 2A and 2B). In general, the pharmacological agents all inhibited early HW-dependent [Ca^2+^]_cyt_ increases and had no effects on late HW-induced [Ca^2+^]_cyt_ variations.

**Figure 3:**
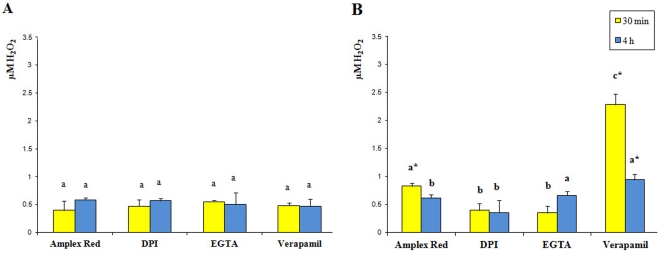
H_2_O_2_ variations in *G. biloba* upon mechanical damage and herbivore wounding. **A**. Mechanically-wounded *G. biloba* leaves, values (n = 5) are expressed as µM H_2_O_2_ calculated from a calibration curve. The same letter indicates not significant (P>0.05) variation. B. Herbivore-wounded *G. biloba* leaves, values (n = 5) are expressed as µM H_2_O_2_. Different letters indicate significant (P<0.05) differences, the asterisk indicate significant (P<0.05) differences with respect to mechanical damage. In both panels, amplex indicates the absence of pharmacological agents.

One of the first reactions to biotic attack is the production of ROS [47]. Hydrogen peroxide (H_2_O_2_) is generated upon herbivore attack in several angiosperms [45]. *G. biloba* fan-shaped leaves showed a significantly higher H_2_O_2_ production 30 min after HW, when compared to MD leaves; however, after 4 h from feeding, HW values dropped to MD levels (Fig. 3). The use of DPI inhibited HW-dependent H_2_O_2_ production that remained at MD levels, and the same was found after 30 min of HW by using EGTA. Verapamil had no effect on MD-dependent H_2_O_2_ production (Fig. 3A) and significantly increased H_2_O_2_ in HW, especially after 30 min of herbivory (Fig. 3B).

**Figure 4:**
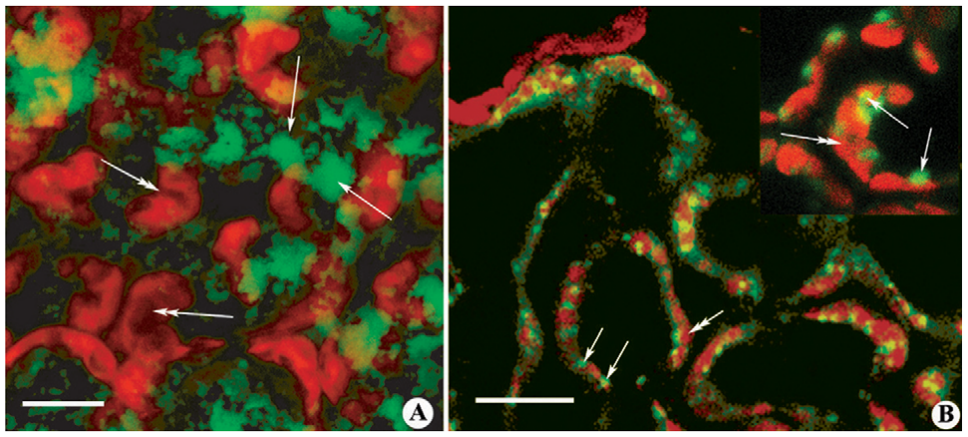
Subcellular localization of [Ca^2+^]_cyt_ and H_2_O_2_ in *G. biloba* leaves upon herbivory. **A**. False color images from confocal laser scanning microscopy shows that upon herbivory [Ca^2+^]_cyt_ was found mainly in the cytosol, indicated by the calcium orange dye as green patches not associated with any specific organelle. Metric bar = 10 µm. **B**. H_2_O_2_ localization by Amplex Red shows a clear associations with microbodies (probably peroxisomes) and/or mitochondria but not with chloroplasts. Metric bar = 20 µm. In both panels, single arrows indicate the dye, double arrows indicate chloroplasts.

The subcellular localization of [Ca^2+^]_cyt_ was found mainly at the cytoplasmic level and was evidenced by the calcium orange dye as patches not associated with specific organelles (Fig. 4A); on the other hand, H_2_O_2_ localization by Amplex Red showed a clear association with microbodies (probably peroxisomes) and/or mitochondria (Fig. 4B).

### Heterologous gene expression analysis of *G. biloba* on Arabidopsis microarray reveals the presence of several conserved defense genes

Analysis of the *G. biloba* transcriptome after herbivory by heterologous microarray hybridization on *Arabidopsis thaliana* genome microarrays revealed the presence of several conserved up- and down-regulated defense genes (see Table S1). Bioinformatic approaches aimed to find orthologous sequences in *G. biloba* ESTs using the oligonucleotide sequences present on the Arabidopsis microarray showed generally a low percentage of sequence identity, with the exception of a protein kinase (At3g01300) (Table S2). Among 146 significantly (fold change ≥2, P≤0.05) modulated genes on the Arabidopsis microarray, we chose 24 genes (17 up and 7 down-regulated) for real-time PCR (qPCR) validation on *G. biloba* cDNA. qPCR confirmed the differential expression for most of these genes, with the exception of some down-regulated genes in the microarray data that were found up-regulated by qPCR, after 4 h of larvae feeding on leaves (Table 1). Most of these genes were associated with biotic and abiotic stress responses. Some were transcription factor regulators: these included a Dof-type zinc finger protein, the phosphate-responsive protein EXO, a MYB transcription factor, and a F-box family protein transcription factor. Other genes, encoding β-galactosidase, guanylate kinase, lipoxygenase, ABC transporter protein, and phospholipase D, are usually involved in plant stress responses. A strong up-regulation was found for a gene (similar to VAMP 724) which encodes a protein that plays a key role in vesicle trafficking to vacuoles and delivery of molecules to their destination. High fold-change expression values were also found for 20S proteasome alpha subunit PAA2 and a putative cytochrome *b5*. Ubiquinol cytochrome *c* reductase, belonging to the family of reductases specifically acting on diphenols, was up-regulated. Down-regulation was confirmed for a protein kinase similar to Arabidopsis APK1A (Table 1).

**Table 1:** Gene expression of *G. biloba* leaves after 4 h from *S. littoralis* herbivory.

	AGI Code	Description	FC	qRT-PCR FC
**Response to biotic and abiotic stimuli**	At1g72520	Lipoxygenase 4 (LOX4)	2.61	2.76±0.38
**Cell wall**	At4g08950	EXORDIUM (EXO); involved in response to brassinosteroid stimulus	2.33	2.87±0.61
**Kinase activity**	At3g57550	Guanylate kinase (GK-2)	3.10	1.71±0.08
	At3g01300	Protein serine/threonine kinase	−2.88	−2.31±0.01
**Hydrolase activity**	At2g05840	20S proteasome alpha subunit A2 (PAA2)	2.19	7.83±0.53
	At3g52840	Beta-galactosidase 2 (BGAL2). Involved in lactose catabolic process, using glucoside 3-dehydrogenase, carbohydrate metabolic process, lactose catabolic process via UDP-galactose.	2.30	2.46±0.07
	At1g59760	ATP dependent RNA helicase. Involved in N-terminal protein myristoylation	−2.74	2.27±0.03
	At4g35790	Phospholipase D/PLD Delta. Involved in phospolipase metabolism. Mutants are affected in hydrogen peroxide mediated cell death.	2.53	1.74±0.32
	At5g66080	Protein phosphatase 2C family protein/PP2C family protein. Protein serine/threonine phosphatase activity	2.45	1.37±0.27
**Transporter activity**	At4g15780	Vesicle-Associated Membrane Protein 724 (VAMP 724). transport, involved in vesicle-mediated transport.	2.09	8.07±0.47
	At2g01090	Ubiquinol Cytochrome C reductase hinge protein; mitochondrial electron transport, ubiquinol to cytochrome c	5.25	7.81±1.05
	At1g09270	Importin alpha isoforms 4 (IMPA-4)	−2.01	3.27±0.24
	At1g01620	Plasma membrane intrinsic protein 1C (PIP1C)	−2.16.	2.56±0.13
	At5g19410	ABC-2 type transporter family protein	2.12	1.14±0.10
**Protein binding**	At2g32720	Cytochrome B5 isoform B	2.31	6.67±1.18
	At4g25340	FKBP-type immunophilin family that functions as a histone chaperone. Binds to 18S rDNA and represses its expression.	2.12	2.66±0.63
**RNA binding**	At3g49390	RNA binding protein (RPB37)	2.44	1.59±0.17
**Transcription factors**	At5g53200	TRIPTYCHON (TRY), Myb transcription factor	−2.17	5.42±1.56
	At5g59570	Brother Of lux Arrhythmo (BOA), a component of the circadian clock. Transcription factor.	−2.63	3.99±0.03
	At5g60850	Dof- type Zinc finger domain similar to zinc finger protein OBP4 transcription factor	3.24	2.27±0.65
	At5g11060	KN1-like homeodomain transcription factor (KNAT4)	−14.00	−1.01±0.01
**Protein metabolism**	At5g43640	Ribosomal protein S19 family protein	2.40	1.36±0.17
**Unkn. biological process**	At2g03505	Carbohydrate-binding X8 domain superfamily protein	9.62	1.88±0.09
**Other metabolic process**	At5g44620	Cytochrome p450 family protein (CYP706A3)	2.61	1.10±0.11

Data are expressed as fold change by considering gene expression in mechanically damaged leaves equal to 1. Microarray data from the heterologous hybridization performed on Arabidopsis microarrays are listed along with qPCR data using *G. biloba* cDNAs. Genes are grouped by GO annotations. (±SD). Microarray Fold Change (FC).

**Figure 5:**
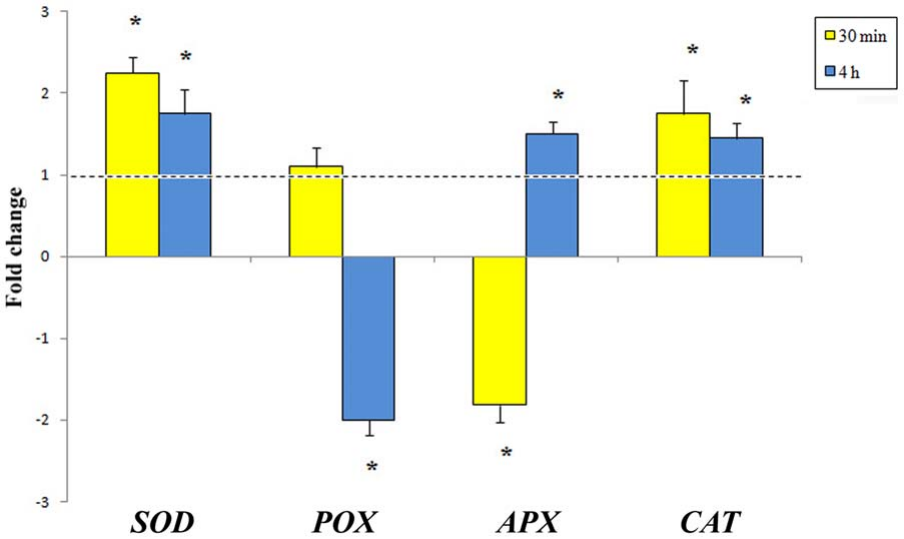
Time-course quantitative gene expression of some ROS scavenging genes in *G. biloba* upon herbivory. Gene expression of superoxide dismutase (SOD) and catalase (CAT) was up-regulated by herbivory at all times. Upon herbivory, peroxidase (POX) was significantly down-regulated after 4 h, whereas ascorbate peroxidase (APX) was down-regualted after 30 min. The dotted lines represent control values (mechanical damage), different letters indicate significant (P<0.05) differences, asterisk indicates significant (P<0.05) differences with respect to control.

Considering the indication that ROS levels are modulated upon feeding, as shown by confocal laser scanning microscopy (CLSM), we extended our gene expression study to four genes coding for ROS-scavenging enzymes: superoxide dismutase (*SOD*), peroxidase (*POX*), ascorbate peroxidase (*APX*) and catalase (*CAT*), following MD and HW treatment (Fig. 5). With respect to MD (dotted line), *SOD* and *CAT* were up-regulated at both time points, whereas *POX* and *APX* showed opposing trends: *POX* was up-regulated at 30 min and down-regulated at 4 h, whereas *APX* was down-regulated at 30 min and up-regulated at 4 h (Fig. 5).

### Herbivory induces the regulation of *G. biloba* direct defenses: flavonoid biosynthesis and gene expression

**Figure 6:**
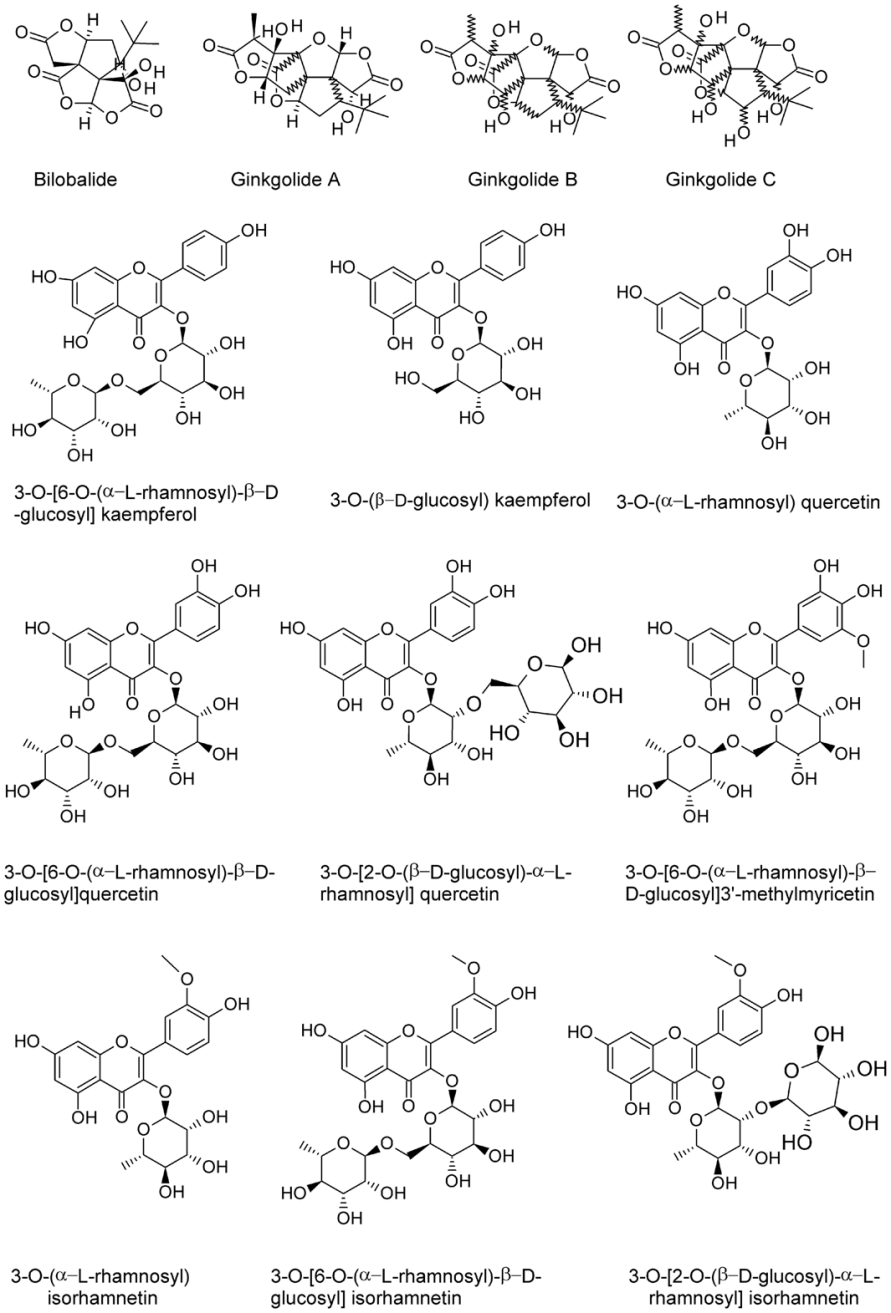
Structure formulae of the main representative *G. biloba* compounds.

*G. biloba* leaves are characterized by the presence of several secondary metabolites, including the terpenoids ginkgolide A, B and C, and bilobalide, and several glycosylated flavonoids (Table 2). Analysis of MD and HW *G. biloba* leaves revealed that the main flavonoid backbones present were quercetin, kaempferol, myricetin and isorhamnetin, which were glycosylated in position 3 by β-d-glucose and α-l-rhamnose (Fig. 6). Chemical analyses were performed 4 h after both MD or HW, which was considered a time long enough to identify trends in metabolic adaptations to insect feeding.

**Table 2:** Comparative analysis of flavonoids, bilobalide and ginkolides between mechanically damaged (MD) and *Spodoptera littoralis* wounded (HW) *Ginkgo biloba* leaves after 4 h feeding.

Compound	Spectra [M-H]^−^	MD	HW
Quinic acid	MS:190.8; MS^2^[190.8]: 173; 127; 111; 93; 85	3.25 ( 1.81)	3.81 ( 0.48)
3-O-[2-O, 6-O-Bis(α-l-rhamnosyl)-β-d-glucosyl]quercetin	MS:755; MS^2^[755]: 609; 301	1.01 ( 0.69)	1.66 ( 0.17)
3-O-[6-O-(α-l-rhamnosyl)-β-d-glucosyl]myricetin	MS:625; MS^2^[625]: 317 MS^3^[317]: 288; 271; 179	0.51 ( 0.38)	1.60 ( 0.53)
3-O-(β-d-glucosyl)quercetin	MS:463; MS^2^[463]: 301	1.78 ( 1.34)	5.66 ( 1.12)
3-O-[6-O-(α-l-rhamnosyl)-β-d-glucosyl]isorhamnetin	MS:623; MS^2^[623]: 315	18.80 ( 5.35)	**49.34** ( 0.78)
Glucosyl myricetin	MS:625; MS^2^[625]: 317 MS^3^[317]: 288; 270; 179	nd	1.44 ( 0.21)
3-O-[2-O-(β-d-glucosyl)-α-l-rhamnosyl]isorhamnetin	MS:623; MS^2^[623]: 315 MS^3^[315]: 301; 272; 255	8.64 ( 2.82)	**16.01** ( 1.85)
3-O-[6-O-(α-l-rhamnosyl)-β-d-glucosyl]quercetin	MS:609.1; MS^2^[609]: 301	20.42 ( 2.38)	**39.09** (10.19)
3-O-[6-O-(α-l-rhamnosyl)-β-d-glucosyl]-3′-methylmyricetin	MS:639.1; MS^2^[639]: 331 MS^3^[331]:316; 289; 271	3.05 ( 1.31)	**5.27** ( 0.44)
3-O-(α-l-rhamnosyl)isorhamnetin	MS:463; MS^2^[463]: 315 MS^3^[315]:301	46.10 (14.89)	**93.44** ( 0.13)
3-O-[2-O-(β-d-glucosyl)-α-l-rhamnosyl]quercetin	MS:609.1; MS^2^[609]: 301 MS^3^[301]:271;	24.11 ( 9.37)	**59.12** ( 4.96)
3-O-[6-O-(α-l-rhamnosyl)-β-d-glucosyl]kaempferol	MS:593; MS^2^[593]:285 MS^3^[285]: 257; 229	12.08 ( 2.86)	**30.22** ( 6.67)
3-O-[2-O-(β-d-glucosyl)-α-l-rhamnosyl]-3′-methylmyricetin	MS:639; MS^2^[639]: 331 MS^3^[331]:316; 287; 271	1.57 ( 0.83)	2.45 ( 0.08)
Diglucosyl isorhamnetin	MS:623.1; MS^2^[623]: 315 MS^3^[315]: 300; 271; 255	14.10 ( 1.95)	**19.62** ( 0.28)
Glucosyl quercetin	MS:463; MS^2^[463]: 301	51.49 ( 5.59)	56.73 ( 2.44)
3-O-[2-O-(β-d-glucosyl)- α-l-rhamnosyl]kaempferol	MS:593; MS^2^[593]:285	nd	**7.72** ( 1.95)
3-O-(β-d-glucosyl)kaempferol	MS:447; MS^2^[447]: 285 MS^3^[285]: 255, 227, 151	2.34 ( 0.88)	**6.42** ( 0.52)
3-O-(α-l-rhamnosyl)quercetin	MS:447; MS^2^[447]: 301 MS^3^[301]: 179; 151	26.46 ( 2.52)	**50.94** ( 9.78)
7-O-(α-l-rhamnosyl)kaempferol	MS:431; MS^2^[431]: 285; 227	19.01 ( 2.83)	22.90 ( 4.45)
Bilobalide	MS:325; MS^2^[325]: 251; 207; 193; 163	265.28 (15.02)	233.03 (15.77)
Ginkgolide A	[M^+^CO_2_]^−^ 453; MS^2^[453]:407; 379; 351	318.47 ( 9.31)	**239.87** (17.69)
Ginkgolide B	MS:423; MS^2^[423]: 395; 367	404.22 (24.76)	**279.91** ( 8.43)
Ginkgolide C	MS:439; MS^2^[439]: 411; 383; 321	65.74 ( 8.17)	**44.60** ( 3.09)

nd, not detected.

Values (n = 5–8) are expressed as ng g^−1^ fr. wt. (±SEM). In the same row, boldface HW values indicate significant (P<0.05) differences between HW and mechanically damaged (MD) leaves.

With respect to MD, HW prompted an almost two fold increase in several glycosylated flavonoids, particularly 3-O-(β-d-glucosyl)kaempferol (2.74-fold, P<0.01), 3-O-[6-O-(α-l-rhamnosyl)-β-d-glucosyl]isorhamnetin (2.6-fold, P<0.05), 3-O-[6-O-(α-l-rhamnosyl)-β-d-glucosyl]kaempferol (2.50-fold, P<0.05) and 3-O-[2-O-(β-d-glucosyl)-α-l-rhamnosyl]quercetin (2.46-fold, P<0.05). HW induced the synthesis of two new compounds: glycosyl myricetin and 3-O-[2-O-(β-d-glucosyl)-α-l-rhamnosyl]kaempferol. Surprisingly, no significant differences were found between HW and MD for one of the most bioactive compounds of *G. biloba*, bilobalide, whereas ginkgolides A, B and C were significantly reduced by HW treatment with respect to MD (−1.33-fold, P<0.05; −1.44-fold, P<0.05 and; −1.47-fold, P<0.05, respectively) (Table 2 and Fig. 6). Control analyses performed on intact leaves showed no significant differences with respect to MD (data not shown).

**Figure 7:**
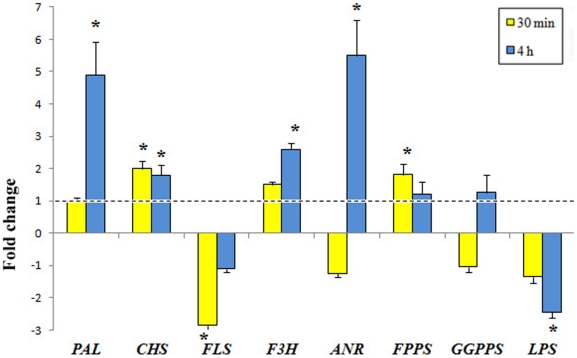
Time-course quantitative gene expression of some *G. biloba* genes involved in phenylpropanoid and terpenoid metabolism upon herbivory. Phenylalanine ammonia lyase (*PAL*) and anthocyanidin reductase (*ANR*) were significantly up regulated by herbivory only after 4 h, whereas flavonol synthase (*FLS*) was down-regulated at 30 min and 4 h. Chalcone synthase (*CHS*) showed a constant up-regulation, whereas flavanone 3-hydroxylase (F3H) showed an increased up regulation after 4 h. Farnesyl diphosphate synthase (*FPPS*) was significantly upregulated only after 30 min whereas geranylgeranyl diphosphate synthase (*GGPP*) showed no regulation at all times. Levopimaradiene synthase (*PPS*) was significantly down-regulated at all times. The dotted lines represent control values (mechanical damage), different letters indicate significant (P<0.05) differences, asterisks indicate significant (P<0.05) differences with respect to control.

We then measured the expression levels of some genes related to phenylpropanoid and terpenoid biosynthesis, respectively, since these compounds are modulated by *G. biloba* responses to HW. Chalcone synthase (*CHS*), which catalyzes the first committed step in flavonoid biosynthesis, was induced comparably at 30 min and 4 h, whereas phenylalanine ammonia lyase (*PAL*), flavanone 3-hydroxylase (*F3H*), and anthocyanidin reductase (*ANR*) were significantly up-regulated by HW only after 4 h. In contrast, flavonol synthase (*FLS*) was down-regulated at both time points (Fig. 7). In *G. biloba*, the universal sesquiterpene precursor farnesyl diphosphate (FPP) is synthesized from geranyl diphosphate by the enzyme FPP synthase (FPPS), whereas the diterpene precursor geranylgeranyl diphosphate (GGPP) is synthesized from isopentenyl diphosphate and FPP by the enzyme GGPP synthase (GGPPS) [8]. Ginkgolide biosynthesis is initiated by protonating GGPP to give labdadienyl diphosphate, then the allylic diphosphate ionization is followed by cyclization, 1,4 hydride shift, methyl migration, and deprotonation to yield levopimaradiene. Levopimaradiene synthase (LPS) catalyzes the initial cyclization step in ginkgolide biosynthesis [48]. A transient up-regulation of *FPPS* was observed after 30 min of herbivory, which dropped back to control levels at 4 h (Fig. 7). *GGPPS* was not significantly regulated by herbivory, whereas a significant decrease of *LPS* expression was observed with time (Fig. 7)

### Herbivory induces *G. biloba* VOCs emission

Although *G. biloba* reacts to herbivory by inducing potentially toxic defense compounds, the plant also emits VOCs (Table 3). We analyzed the composition and quantity of VOCs by Tenax TA adsorption and GC-MS analysis of the headspace of treated leaves after 4 h and 24 h and found a significantly (P<0.05) higher emission after infestation by *S. littoralis* in comparison to mechanical damage (Table 3).

**Table 3:** Analysis of VOCs in the headspace of treated *G. biloba* leaves.

		4 h		24 h	
Compounds	KI	MD	HW	MD	HW
2-Methyl butane	454	3.03 (0.26)	**1.02 (0.11)**	0.40 (0.50)	**8.22 (1.73)**
Octane	800	2.93 (0.91)	1.64 (1.11)	3.86 (0.31)	3.38 (0.29)
2-Hexenal	855	7.74 (4.40)	5.06 (3.71)	1.91 (2.53)	**8.23 (0.75)**
Heptanal	902	6.25 (2.19)	7.04 (2.77)	7.66 (2.34)	5.10 (3.21)
2-Heptenal	964	5.57 (0.37)	**13.11 (2.10)**	5.09 (0.84)	**9.62 (0.55)**
2-Penthyl furan	992	9.92 (1.15)	10.73 (2.97)	5.28 (1.48)	5.40 (0.54)
Decane	1000	7.15 (1.36)	6.23 (2.50)	7.31 (2.32)	5.90 (1.36)
Octanal	1004	2.30( 0.90)	3.46 (1.33)	1.34 (0.13)	0.91 (0.56)
Limonene	1029	18.71( 2.34)	20.25 (2.55)	4.42 (2.90)	12.10 (0.48)
2-Octenal	1056	10.79 (0.78)	**6.76 (1.08)**	4.07 (0.85)	3.40 (1.36)
1-Octanol	1068	6.38 (1.47)	**19.28 (1.16)**	2.78 (0.07)	**4.33 (0.91)**
Nonanal	1101	0.28 (0.11)	0.45 (0.27)	0.14 (0.02)	0.10 (0.01)
2-Nonenal	1149	6.74 (2.40)	7.43 (1.52)	3.83 (0.39)	**10.41 (1.65)**
Ethyl benzoate	1173	28.14 (7.48)	29.92 (2.61)	1.29 (0.16)	**6.76 (1.83)**
Decanal	1202	0.73 (0.13)	0.65 (0.52)	0.43 (0.17)	0.13 (0.11)
Benzothiazole	1218	2.97 (0.95)	3.60 (1.53)	0.86 (0.69)	0.43 (0.06)
2-Decenal	1249	6.59 (1.68)	8.01 (3.54)	1.58 (0.43)	1.53 (0.24)
Undecanal	1307	5.03 (1.81)	4.62 (2.91)	2.38 (0.59)	1.27 (0.93)
α-Copaene	1377	1.09 (0.67)	**6.58 (0.53)**	4.59 (2.77)	**11.89 (0.22)**
1-Tetradecene	1390	7.43 (1.77)	8.44 (2.69)	11.00 (2.62)	11.39 (2.58)
Tetradecane	1400	5.95 (0.38)	**8.50 (0.79)**	1.91 (0.42)	1.84 (0.39)
Dodecanal	1402	8.48 (1.63)	9.19 (2.87)	4.33 (2.87)	2.90 (1.70)
(*E*)-β-caryophyllene	1419	5.25 (1.77)	**24.26 (1.52)**	2.74 (0.69)	**8.30 (1.70)**
β-Chamigrene	1453	2.67 (0.60)	**12.16 (1.82)**	6.37 (0.99)	6.25 (1.63)
Pentadecane	1478	1.49 (1.14)	1.90 (0.91)	0.58 (0.10)	0.43 (0.09)
Total		198.14 (11.84)	**250.02 (12.63)**	97.91 (18.60)	**153.07 (26.64)**

Data are expressed as micrograms of VOCs per gram of leaf fresh weight (±SEM). Retention times (RT) and Kováts Index (KI) are indicated for each compound. For the same time point, boldface HW values indicate significant (P<0.05) differences between MD and HW. HW, herbivore wounding; MD, mechanical damage.

After 4 h feeding by *S. littoralis*, the emission of 1-octanol (3-fold), 2-heptenal (2.4-fold) and the sesquiterpenes α-copaene (6-fold) and β-caryophyllene (4.6-fold) was always significantly (P<0.05) higher in HW with respect to MD. The green leaf volatile (GLV) 2-hexenal did not show significant changes, whereas the emission of 2-octenal was significantly higher in MD.

After 24 h, the emission of 2-methyl butane increased significantly in HW plants with respect to MD (Table 3). A significant increase was also observed in HW for the two GLVs, 2-hexenal and 2-heptenal. In HW leaves, the emission of 1-octanol was still significantly enhanced, and a significant increase was found for 2-nonenal and ethyl benzoate. The emission of the two sesquiterpenes α-copaene (2.6-fold) and β-caryophyllene (3-fold) was still higher in HW in comparison to MD, although to a lesser extent with respect to 4 h time point.

## Discussion

Plants and insects have coexisted for as long as 350 million years, if the earliest forms of land plants and insects are included, and have developed a series of relationships affecting the organisms at all levels, from basic biochemistry to population genetics. Although some of the relationships between the two kingdoms, such as pollination, are mutually beneficial, the most common interaction involves insect folivory and plant direct and indirect defenses against herbivorous insects [35], [49], [50]. On the basis of this long-standing relationship, it is not surprising that the strategies used by plants to resist or evade insect herbivores may be based on a common strategy. Although some species accumulate high levels of toxic compounds which function as direct biochemical defenses, other may not commit resources for this strategy, but seek to minimize herbivore damage through rapid growth and development, dispersion, choice of habitat, or by emitting VOCs able to attract enemy's enemies [11], [36], [51]. Despite this diversity, our study on *G. biloba* shows that there is a general common defensive mechanism for plant response to herbivore wounding.

Our results show that the so called “living fossil plant” *G. biloba* uses early and late responses which are comparable to those found in angiosperms [13], [14], [40], [52]. This is not surprising, since lower plants like the fern *Pteris vittata* have been shown to respond to herbivory by ROS production and the emission of volatile compounds [53]. In *G. biloba*, Vm variations, although small, were significantly different between HW and MD, indicating that also in this species early detection of herbivory involves an ion imbalance across the plasma membrane [40] and possibly the perception of insect elicitors by plant cell receptors. In angiosperms such as Lima bean, Vm variations are associated to changes in calcium homeostasis [46]. *G. biloba* leaves reacted to HW with a burst of [Ca^2+^]_cyt_, that was inhibited by the use of the calcium chelator EGTA and the inward calcium channel inhibitor Verapamil, as found in angiosperms [13], [41]. Surprisingly, DPI also inhibited HW-induced [Ca^2+^]_cyt_, suggesting an interplay between H_2_O_2_ and calcium homeostasis [45]. In fact, the use of Verapamil induced a significant burst of H_2_O_2_, whereas EGTA reduced H_2_O_2_ production. The subcellular localization of calcium and H_2_O_2_ signaling upon HW were in the cytosol and mitochondria/peroxisomes, respectively, as already observed in angiosperms [45], [46], [52].

Upon HW, several genes were differentially expressed with respect to MD. The observed increase in H_2_O_2_ was in accordance with the increased transcript levels of *SOD* and *CAT* at all time points, as previously found upon herbivory in the model plant Lima bean [45]. On the other hand *POX* was found to be significantly down regulated by herbivory at later times, whereas *APX* was down-regulated at early times. The down-regulation of *POX* has been associated to the effect of insect's oral secretions [54].

The results obtained with our heterologous microarray experiment showed that most of the modulated genes were associated with biotic and abiotic stress responses. A strong up-regulation was found for a gene encoding a protein with transporter activity, the Vesicle-Associated Membrane Protein 724 (*VAMP 724*, v-SNARE). This protein forms a complex known as SNARE (soluble *N*-ethylmaleimide-sensitive-factor attachment protein receptor), that plays a key role in vesicle trafficking to vacuoles and delivery of molecules to their destination. The major role carried out by this protein is to move ROS from endosomes to vacuoles. Suppression of *Arabidopsis* vesicle *VAMP 724* expression inhibits fusion of H_2_O_2_ containing vesicles with vacuoles [55]. Another up-regulated gene with transporter activity is ubiquinol cytochrome *c* reductase (cytochrome *bc1* complex or complex III). The activity of the gene product is involved in mitochondrial ROS production, particularly H_2_O_2_, which acts not only as a damaging oxidant but also as a signaling molecule through either direct (oxidation of its target) or indirect (e.g., involving peroxiredoxins) action [56]. Interestingly, the 20S proteasome alpha subunit *PAA2* proved to be highly induced by herbivory. Dahan and co-workers [57] hypothesized a complex organization and regulation of the 20S plant proteasome and its possible stress-induced modification into a so-called “plant defense proteasome”, which might be involved in the activation of plant defense reactions. The same authors also demonstrated that 20S proteasome alpha subunit is up regulated by elicitins in tobacco cells. Other up-regulated genes involved in transport processes were *importin á* (*IMPA-4*), one of the two factors of the nuclear pore-targeting complex which was found to interact with virulence (Vir) proteins encoded by the Ti plasmid of *Agrobacterium tumefaciens* [58]; and an aquaporin (*PIP1c*), which is involved in water transport activity, and which has been recently correlated to ROS signaling and/or oxidative stress response [59].

Upon herbivory cytochrome *b5* was also up-regulated. Cytochrome *b5* is a heme-binding protein and functions as an electron transfer component involved in a number of oxidative reactions, such as the anabolic metabolism of lipids and the catabolism of xenobiotics and compounds of endogenous metabolism [60]. The oxidative reactions mediated by cytochrome *b5* are also associated with sugar supply and cytochrome *b5* plays a regulatory role by physically interacting with sugar transporters [61].

The gene encoding for a protein serine/threonine kinase, similar to protein kinase APK1A, was found to be down regulated upon herbivory. The involvement of protein kinases in plant-herbivore interaction has been recently reviewed [14].

Two transcription factors, *TRIPTYCHON* (*TRY*) and a component of the circadian clock (*BROTHER OF LUX ARRHYTHMO*, *BOA*), showed a consistent up-regulation. *TRY*, which encodes a CPC-homologous MYB-related transcription factor, is a negative regulator of trichome development functioning in lateral inhibition and hence most probably in cell-cell signaling [62]. *BOA* is a GARP family transcription factor and is regulated by circadian rhythms in *A. thaliana*. Overexpression of *BOA* exhibits physiological and developmental changes, including delayed flowering time and increased vegetative growth under standard growing conditions [63].

Phenolic compounds are apparently important in the defense mechanisms of conifers [64] and the induction of leaf flavonoids is a specific defense response of many plants against insect herbivory [65], [66]. Our results on *G. biloba* flavonoid metabolism and gene expression indicate an involvement of flavonoids in response to herbivory. *PAL*, *CHS*, *F3H* and *ANR* gene expression were up-regulated after 4 h. This increased gene expression was accompanied by the increased abundance of several flavonoids like 3-O-(β-D-glucosyl)kaempferol, 3-O-[6-O-(α-L-rhamnosyl)-β-D-glucosyl]kaempferol, 3-O-[2-O-(β-D-glucosyl)-α-L-rhamnosyl]kaempferol and 3-O-[6-O-(α-L-rhamnosyl)-β-D-glucosyl]isorhamnetin. Kaempferol diglycoside, kaempferol triglycoside, and quercetin glycosides were also found to be significantly increased by beetle damage [67]. In *G. biloba*, 3-O-[2-O-(β-D-glucosyl)-α-L-rhamnosyl]quercetin was also increased after 4 h herbivory, although the gene expression of a flavonol synthase (*FLS*), which leads to the synthesis of quercetin from dihydroflavonols, was down-regulated.

Although increased contents of bilobalide, ginkgolide A and ginkgolide B have been observed in *G. biloba* cell cultures induced with biotic elicitors of *Candida albicans* [68] or in plants exposed to elevated levels of O_3_ [69], the concentration of bilobalide did not show significant changes in response to herbivory, whereas the ginkgolides A, B and C were all significantly reduced by insect feeding. Ginkgolide B has been shown to confer bioactivity by inhibiting oxidative stress generation, and the dose-response effects of ginkgolide B on ROS generation in human cells has been demonstrated [70]. We may speculate that the increased ROS activity upon HW may exert a negative effect on ginkgolide production. The observed down-regulation of *LPS* at all times correlated with the reduction of ginkgolide content.

Herbivory also induced *G. biloba* VOCs emissions. Previous attempts to induce VOC emission from *G. biloba* with spider mites (*Tetranychus urticae*) were unsuccessful, because leaves were not accepted as a host plant. However, treatment of *G. biloba* leaves with 1 mM jasmonic acid (JA) induced VOC emission [31], suggesting the potential of this plant to emit terpenoids. The generalist *S. littoralis* accepted *G. biloba* leaves although feeding induced a delay in molting and the death of some insect (unpublished results). Herbivore-induced VOCs included the sesquiterpenes α-copaene and β-caryophyllene, which have been shown to be released by *G. biloba* upon JA treatment [31]. These two sesquiterpenes have also been described to be involved in attraction of insect's predators in several plant-interactions [51]. Finally the increased emission of some HW-induced sesquiterpenes was accompanied by the up-regulation of *FPPS*, a key gene in sesquiterpene synthesis [71]. The conversion of FPP to the sesquiterpenes α-copaene [72] and β-caryophyllene [73] has been demonstrated.

In conclusion, we showed that the “living fossil” plant *G. biloba* responds to herbivory by inducing early responses, such as the variation of the plasma transmembrane potential and the induction of both calcium and ROS signaling. These events preceded the activation of “second line” defense systems including the activation of defense genes and the production of secondary plant metabolites (e.g., many glycosylated flavonoids). Furthermore the emission upon herbivory of specific VOCs indicates the ability of the plant to potentially activate indirect defenses along with the activation of direct defenses, although the ability of emitted VOCs to attract predators of herbivores was not yet demonstrated. Current research in our laboratory is under way to evaluate the possible attraction of predators by emitted VOCs as well as *S. littoralis* tolerance to *G. biloba* toxic metabolites and the mechanisms underlying its resistance.

## Materials and Methods

### Plant and animal material

*Ginkgo biloba* L. seeds were collected from an adult (>100 years old) female *G. biloba* tree growing in the Botanical Garden of the University of Turin. Seeds were germinated in plastic pots with sterilized potting soil at 27°C during the day and 22°C during the night and 60% humidity using daylight fluorescent tubes at approximately 120 µmol m^−2^ s^−1^ with a photoperiod of 16 hours. Experiments were conducted with three-month old plants on fully developed leaves which were found to be the most responsive leaves.

Larvae of the generalis herbivore *Spodoptera littoralis* (Boisd. 1833) (Lepidoptera, Noctuidae) (kindly supplied as egg clutches by Dr. Roland Reist, Syngenta Crop. Protection Münchwilen AG, Stein, Switzerland), were used because to our knowledge there are no reports on herbivores feeding on *G. biloba*. Larvae were reared in Petri dishes at 22–24°C with a 14–16 h light phase. They were fed on artificial diet as previously described [46].

All experiments, except preliminary Vm tests on bilobed and multi-dissected leaves, were carried out by using *G. biloba* fan-shaped leaves at the same developmental stage. *S. littoralis* larvae (third instar) were starved for 24 h before transfer to leaves. The mechanical damage was done by a pattern wheel. Damaged leaves were harvested by cutting the petiole and immediately frozen in liquid nitrogen and stored at −80°C until use.

### Membrane potentials

Membrane potentials were determined in leaf segments. *G. biloba* bilobed, fan-shaped and multi-dissected leaves were analyzed. The transmembrane potential difference (Vm) was determined as previously reported [46]. Vm variations were recorded both on a pen recorder and through a digital port of a PC using a data logger. The results of Vm are shown as the average number of at least 50 Vm measurements.

### Determination of intracellular calcium variations using confocal laser scanning microscopy (CLSM) and calcium orange

Calcium orange dye (stock solution in DMSO, Molecular Probes, Leiden, The Netherlands) was diluted in 5 mM MES-Na buffer (pH 6.0) containing 0.5 mM calcium sulfate and 2.5 µM, dichlorophenyldimethylurea (DCMU) (Sigma-Aldrich, Milan, Italy) to a final concentration of 5 µM. This solution was applied on *G. biloba* fan-shaped leaves attached to the plant. The leaf was gently fixed on a glass slide and a drop of 5 µM calcium orange solution (about 45 µl) was applied and covered with another glass slide. After one hour of incubation with calcium orange, the leaf was mounted on a Nikon Eclipse C1 (Nikon Instruments, Tokyo, Japan) spectral CLSM stage without separating the leaf from the plant in order to assess the basic fluorescence levels as a control. Calcium variations were also monitored following MD and HW for 30 min and 4 h in the presence of either 15 µM diphenyleneiodonium (DPI; Sigma-Aldrich), 100 µM Verapamil (Fluka Biochemika, Buchs, Switzerland) or 250 µM ethylene glycol-bis(2-aminoethylether)-N,N,N′,N′-tetraacetic acid (EGTA, Sigma-Aldrich).

The microscope operated with a Krypton/Argon laser at 543 nm and 568 nm wavelengths: the first wavelength excited calcium orange, resulting in green fluorescence and the second mainly excited chlorophyll, resulting in red fluorescence. Images generated by the FluoView software were analyzed using the NIH image software as described earlier [41]. Measurements were repeated at least 5 times (biological replicates).

### CLSM localization of H_2_O_2_ and active peroxidases using 10-acetyl-3,7-dihydroxyphenoxazine (Amplex Red)

The amplex red hydrogen peroxide/peroxidase assay (Molecular Probes) was used for the detection of H_2_O_2_ and active peroxidases. *G. biloba* fan-shaped leaves from intact plants in pots were incubated with a 50 µM amplex red solution (in 5 mM Mes-Na buffer, pH 6.0, containing 0.5 mM calcium sulfate and 5 µM DCMU) as reported earlier [45]. Leaves were mounted on a Nikon Eclipse C1 spectral CLSM stage without separating the leaf from the plant in order to determine the background fluorescence. H_2_O_2_ variations were monitored after MD or HW treatment for 30 min and 4 h, and in addition in the presence of either 15 µM DPI, 100 µM Verapamil or 250 µM EGTA. The microscope was operated with an Ar-Laser (458 nm/5 mW; 476 nm/5 mW; 488 nm/20 mW; 514 nm/20 mW), a HeNe-Laser (543 nm/1,2 mW) and a HeNe-Laser (633 nm/10 mW). Measurements were repeated at least 5 times (biological replicates).

### Isolation of total RNA and cDNA synthesis

Total RNA was extracted from treated (HW) and control (MD) *G. biloba* leaves by using the Agilent Plant RNA Isolation Mini Kit (Agilent Technologies), following manufacturer's instructions. To remove residual genomic DNA, total RNA was treated with RNAse-free DNAse I set (Qiagen, Hilden, Germany) The RNA quality was checked using the Agilent 2100 Bioanalyzer on RNA 6000 Nano LabChips Kit (Agilent Technologies). Quantitative analysis was performed using the NanoDrop ND-1000 micro scale spectrophotometer (Thermo Fisher Scientific, Waltham, MA, US) as previously reported [15].

For cDNA synthesis, High-capacity cDNA Reverse Transcription Kit (Applied Biosystems) was used according to manufacturer's instructions. Briefly, the reactions were prepared by adding 1.5 µg total RNA, 2 µl of 10× RT buffer, 0.8 µl of 25× dNTPs mix (100 mM), 2 µl 10× RT random primer, 1 µl of Multiscribe™ reverse transcriptase and nuclease-free sterile water up to 20 µl. Then the reaction mixtures were incubated at 25°C for 10 minutes, 37°C for 2 hours, and 85°C for 5 seconds. Samples were stored at −20°C for further analyses.

### Heterologous gene microarray hybridization

Five hundred nanograms of total RNA from MD and HW-treated samples were separately reverse-transcribed into double-strand cDNAs by the Moloney murine leukemia virus reverse transcriptase (MMLV-RT) and amplified for 2 h at 40°C using the Agilent Low RNA Input Linear Amplification Kit, two-color (Agilent Technologies, Santa Clara, CA, US). Subsequently, cDNAs were transcribed into antisense cRNA and labeled with either Cy3-CTP or Cy5-CTP fluorescent dyes for 2 h at 40°C following the manufacturer's protocol. Cyanine-labeled cRNAs were purified using RNeasy Minikit (Qiagen, Hilden, Germany). Purity and dye incorporation were assessed by spectrophotometry and electrophoresis (using the NanoDrop ND-1000 and Agilent 2100 Bioanalyzer LabChips, respectively). Then, 750 ng of Cy3-labeled RNA of the control condition and 750 ng of Cy5-labeled RNA of the experimental condition (HW) were combined and hybridized using the Gene Expression Hybridization Kit (Agilent Technologies) onto 1×22 K Arabidopsis (v2) Oligo Microarray (Agilent Technologies).

After a 17 h incubation at 65°C and 10 rpm, the microarray was first washed with gene expression wash buffer 1 for 1 min, then with gene expression wash buffer 2 for 1 min, then with 100% acetonitrile for 30 s, and finally washed in the stabilization and drying solution for 30 s.

The microarray slide was scanned with the Agilent Microarray G2505B Scanner and data were extracted and normalized from the resulting image using Agilent Feature Extraction (FE) software (v.9.5.1). Data were analyzed using the GeneSpring GX 10.1.1 software (Agilent Technologies).

### Bioinformatics analyses

About 4300 EST sequences isolated from *G. biloba* female leaf were downloaded from the National Centre for Biotechnology Information (NCBI) database (http://www.ncbi.nlm.nih.gov/). BlastX analyses were carried out using the NCBI blast tool and The Arabidopsis Information Resource (TAIR) database (http://www.arabidopsis.org/), i) in order to identify *G. biloba* genes with similarity to those oligonucleotide probes on the microarray which were found to be differentially expressed by cross-hybridization, and ii) in order to predict potential protein functions for these genes. Twenty four genes, out of the almost 100 genes which were significantly modulated in the microarray, were selected and validated by qPCR.

In order to find genes encoding enzymes involved the biosynthesis of phenylpropanoids and in the ROS scavenging system to be employed in expression analyses, an additional search in NCBI (EST database) was carried out.

### Quantitative real time PCR (qPCR)

qPCR analyses were carried out using the Stratagene MPX3000 Real Time System (La Jolla, CA, USA). qPCR reactions were run using specific primers designed with Primer3 software (http://frodo.wi.mit.edu/primer3/) and listed in Supporting Table S3 and *G. biloba* cDNAs as template. Amplifications were carried out in a 25 µl reaction mixture containing 1 µl cDNA as template (1∶10 dilution of cDNA from 20 µl of RT reaction), 12.5 µl Maxima™ SYBR Green qPCR master mix (2×) (Fermentas, International, Inc, Burlington, ON, Canada) and 100 nM primers (Integrated DNA Technologies, Coralville, IA, US). The applied protocol was the following: initial polymerase activation of 10 min at 95°C; followed by 40 cycles of 30 s at 95°C, 30 s at 57°C, and 30 s at 72°C. Fluorescence was read following each annealing and extension phase. All runs were followed by a melting curve analysis from 55 to 95°C. Three different reference (housekeeping) genes (actin 2, glyceraldehyde-3 phosphate dehydrogenase, 18 S rRNA) were used to calibrate and normalize the results of the qPCR. The best of the three genes was selected using the Normfinder software (www.normfinder.com). The most stable gene was actin 2. PCR conditions were determined by comparing threshold values in dilution series of the RT product, followed by non-template control for each primer pair. Relative expression levels of genes were calculated by using the Pfaffl method [74].

### Extraction and analysis of *G. biloba* compounds induced by MD and HW

One gram of frozen leaves was ground to a fine powder by using liquid nitrogen with the addition of 10 ml methanol (Carlo Erba Reagents, Arese, Italy). Samples were extracted in a ultrasonic bath at 35°C for 30 min and centrifuged at 5000 *g* for 10 min at room temperature. The supernatant was transferred and the same extraction procedure was repeated twice. Pooled aliquots were dried under vacuum. Extracts were re-suspended in 500 µl methanol and then centrifuged at 5000 *g* for 10 min at room temperature. Extracts were filtered before injection in LC/MS. Samples were separated by an Agilent 1200 HPLC (Agilent Technologies) equipped with a Luna C18 (3.0×150 mm, 3.0 µm, Phenomenex, Torrance, CA, USA) reversed-phase column. The binary solvent system was: A) double distilled water with 0.1% v/v formic acid and B), acetonitrile (ACN) with 0.1% v/v formic acid. Separation was performed at 0.2 ml min^−1^ flow rate and 25°C using an ACN gradient. The B mobile phase was held at 25% for 3 min and then increased to 30% at 7 min. Isocratic elution was performed for 8 min. Afterwards, solvent B was increased up to 55% (15 to 22 min), and 95% (23 to 27 min). The column was kept at 95% solvent B for 7 min. The initial mobile phase was re-established for 10 min before the next injection.

Mass spectrometry analyses were performed with a 6330 Series Ion Trap LC-MS System (Agilent Technologies) equipped with an electrospray ionization source (ESI). Qualitative analyses were made by tandem MS_3_ and spectra were acquired in negative mode with 1.5 kV ion spray voltage, nebulizer curtain gas (N_2_) at 5 L min^−1^ and 325°C, 1.00 V fragmentation amplitude and full scan range 50–1000 *m/z*. For quantitative analyses, samples were analyzed by LC-ESI-MS_2_ in MRM mode with the above acquisition parameters. The monitored mass transitions were *m/z* 407→351, 423→367, 439→383 and 325→163 for ginkgolides A, B and C and bilobalide, respectively, and the Y_0_^−^ aglycone ions for flavonoid-glycosides. Spectral data were processed and analysed by the DataAnalysis for 6330 Series Ion Trap LC/MS 4.0 software (Bruker Daltonik, Bremen, Germany). Identification of spectra was done by manual interpretation and by comparison with literature data [75], [76]. External calibration curves were made with standard solutions of rutin, quercetin, kaempferol, ginkgolide A, ginkgolide C and bilobalide (Sigma-Aldrich).

### VOC extraction and analysis

Headspace VOCs were collected in 4 L glass desiccators by using four-node cuttings of fan-shaped leaves. Leaves were illuminated with fluorescent light bulbs (70 µmol m^−2^ s^−1^) with a light phase of 16 h, the temperature inside desiccators was 23°C and the relative humidity about 70%. Glass desiccators were connected to a GC grade air generator (HPZA-3500–220, Parker Balston, Cleveland,OH, USA) through a cork plug with two openings. Air was fluxed into the jars at 200 ml min^−1^ flow rate.

Clean glass Thermal Desorption Unit (TDU) liners (Gerstel, Mülheim an der Ruhr, Germany) were filled with 20 mg sorbent Tenax TA 60/80 [poly-(2,6-diphenyl)- p-phenylene oxide] (Supelco, Bellefonte, PA, USA). The sorbent was sandwiched by silanized glass wool (Agilent Technologies). Before use, Tenax TA was always preconditioned at 250°C on a Gerstel TDU for 10 min. Undamaged plants as control, MD-leaves and HW-leaves with six third instar *S. littoralis* larvae were assayed for 4 h and 24 h. All experiments were standardized with 30% of leaf damage.

Tenax TA was desorbed in the TDU connected to a Gerstel Cooled Injection System 3 (CIS3 cryofocusing system) which uses liquid CO_2_ as cooling agent. Desorption was carried out in splitless mode with the following temperature program: 36°C held for 0.5 min, 25°C min^−1^ increase to 260°C. The desorption temperature was held for 5 min and the CIS3 was maintained at −40°C. After desorption, CIS3 temperature was raised at a 12°C sec^−1^ rate up to 280°C and temperature was held for 3 min.

Desorbed volatiles were analyzed by gas-chromatography (Agilent Technologies, mod. 6890N) coupled with a mass spectrometry (Agilent technologies, mod. 5973A). Compounds were separated on a Zebron ZB-5MS (mod. 7HG-G010-11, Phenomenex) capillary column (stationary phase: 95% polydimethyl siloxane - 5% diphenyl, 30 m length, 250 µm internal diameter, 0.25 µm film thickness) with the following temperature program: 60°C for 5 min followed by a temperature rise at a 3°C min^−1^ rate to 270°C (held for 5 min). Tenax TA was exposed in the TDU port during the entire GC run. Carrier gas was He with a constant flow of 1 ml min^−1^, transfer line temperature to MSD was 280°C, ionization energy (EI) 70 eV, and full scan range 50–250 *m/z*. Separated compounds were identified by pure standard comparison, by comparison of their mass spectra and retention indexes (Kováts index) with those of reference substances and by comparison with the NIST mass spectral search software v2.0 using the libraries NIST 98 library. External calibration curves were made with standard solution of (-)-menthol (99%, Fluka) for quantitative measurements as previously described [15].

### Statistical analysis

The overall data sets are expressed as mean values of at least three biological replicates. Three technical replicates were run for each biological replicate. Metric bars indicate standard error. ANOVA and Tukey–Kramer's HSD test (P<0.05) were used to determine significant differences among treatments using the SYSTAT 10 software.

## Supporting Information

Table S1 **List of genes with fold change >2 and P values<0.05 from the microarray heterologous expression along with GO annotation analysis.**
(XLS)

Table S2 **Similarity between** ***Ginkgo biloba*** **EST sequences and** ***Arabidopsis thaliana*** **genes.**
(DOC)

Table S3 **List of primers used in this study.**
(DOC)
