# Supplementary material for: Ginkgo biloba Responds to Herbivory by Activating Early Signaling and Direct Defenses
Source: PLoS One. 2012 Mar 20;7(3):e32822. doi: 10.1371/journal.pone.0032822 (PMC3308967; doi:10.1371/journal.pone.0032822)
Supplement: Table S2 — Similarity between Ginkgo biloba EST sequences and Arabidopsis thaliana genes. (DOC) [file pone.0032822.s002.doc]

**Supplementary Table S2.** Similarity between *Ginkgo biloba* EST sequences and *Arabidopsis thaliana* genes.

| ***A. thaliana* gene** | ***G. biloba* EST** | **Blastx**  **E-Value** | **% similarity** |
| --- | --- | --- | --- |
| Protein kinase, putative similar to protein kinase APK1A (At3g01300) | EX930573 | 2e-62 | 64.0 |
| Cytochrome p450 family protein (At5g44620) | EX931477 | 6e-61 | 46.6 |
| MYB transcription factor (At5g59570) | EX931599 | 2e-25 | 44.8 |
| Synaptobrevin related family protein (At4g15780) | EX931549 | 5e-64 | 43.5 |
| Cytochrome b5, putative similar to Cytochrome B5 (At2g32720) | EX931769 | 9e-19 | 37.9 |
| Plasma membrane intrinsic protein 1C (PIP1C)/aquaporin PIP1.3 (PIP1.3)/ transmembrane protein B (TMPB) (At1g01620) | EX930884 | 2e-79 | 37.9 |
| Immunophilin related/FKBP-type peptidyl-prolyl cis-trans isomerase related Immunophilin FKBP46 (At4g25340) | EX930720 | 3.0 | 33.4 |
| 40S Ribosomal protein RPS15E (At5g43640) | EX930206 | 5e-59 | 33.4 |
| Dof- type Zinc finger domain similar to zinc finger protein OBP4 (At5g60850) | EX930621 | 5e-19 | 30.0 |
| ABC Transporter (At5g19410) | EX931067 | 1e-06 | 29.5 |
| Protein phosphatase 2C family protein / PP2C family protein (At5g66080) | EX931491 | 8e-17 | 28.0 |
| Lipoxygenase (At1g72520) | EX930733 | 2e-80 | 27.5 |
| Guanylate kinase (GK-2) (At3g57550) | EX931690 | 3e-13 | 27.1 |
| Phosphate responsive protein, putative (EXO), similar to Phi-1 (Phosphate induced gene) (At4g08950) | EX930842 | 2e-34 | 25.0 |
| Homeobox protein knotted-1 like 4 (KNAT4) (At5g11060) | EX930623 | 9e-08 | 24.8 |
| 20S proteasome alpha subunit A2 (PAA2) (At2g05840) | EX930285 | 2e-18 | 24.6 |
| Ubiquinol Cytochrome C reductase (At2g01090) | EX931160 | 6e-09 | 23.7 |
| Glycosyl hydrolase family protein (At2g03505) | EX931200 | 5e-19 | 19.0 |
| Beta-galactosidase (At3g52840) | EX931099 | 5e-58 | 17.8 |
| Phospholipase D/PLD Delta  (At4g35790) | EX931456 | 2e-66 | 15.1 |
| F-box family protein (At5g53200) | EX930398 | 7e-50 | 14.0 |
| Importin alpha subunit IMPA4 (At1g09270) | EX931076 | 5e-29 | 13.4 |
| RNA binding protein 37 (At3g49390) | EX931655 | 4e-28 | 11.6 |
| ATP dependent RNA helicase (At1g59760) | EX931761 | 9e-09 | 8.6 |
