# Supplementary material for: Ginkgo biloba Responds to Herbivory by Activating Early Signaling and Direct Defenses
Source: PLoS One. 2012 Mar 20;7(3):e32822. doi: 10.1371/journal.pone.0032822 (PMC3308967; doi:10.1371/journal.pone.0032822)
Supplement: Table S3 — List of primers used in this study. (DOC) [file pone.0032822.s003.doc]

**Supplementary Table S3. List of primers used in this study**

| **MICROARRAY VALIDATION** | | |
| --- | --- | --- |
| **AGI Code and description** | **GenBank Acc.** | **Primers** |
| Synaptobrevin related family protein (At4g15780) | EX931549 | Fwd 5’-ACCTGAGCCTTCACTTTTGCTA-3’  Rev 5’-CCAATGGCCTTTCTGGAG-3’ |
| 20S proteasome alpha subunit A2 (PAA2) (At2g05840) | EX930285 | Fwd 5’-AGACGGGCGTGTATTGTCTC-3’  Rev 5’-TCCAAGATCGTTGTGTGCAG-3’ |
| Ubiquinol Cytochrome C reductase (At2g01090) | EX931160 | Fwd 5’-AGCGGCTAGTGGACTATGGA-3’  Rev 5’-AATCCTCCCCCAAGACAAGA-3’ |
| Cytochrome b5, putative similar to Cytochrome B5 (At2g32720) | EX931769 | Fwd 5’-GCAGTCTTTCCCTTGTACGCC-3’  Rev 5’-TGGGTGCTCTTCCAGAAAC-3’ |
| Phosphate responsive protein, putative (EXO), similar to Phi-1 (Phosphate induced gene) (At4g08950) | EX930842 | Fwd 5’**-**CCAAATAGCCGGTAGCAAGA-3’  Rev 5’-ACGAACCCATTCAACACTGG-3’ |
| Immunophilin related/FKBP-type peptidyl-prolyl cis-trans isomerase related Immunophilin FKBP46 (At4g25340) | EX930720 | Fwd 5’-CAGTCCCACCCAATTCAACTC-3’  Rev 5’-ATAGCCTTCCCCTTCCTTC-3’ |
| Beta-galactosidase (At3g52840) | EX931099 | Fwd 5’-TCAGGAGTTTCCCTGGTGAC-3’  Rev 5’-GCACATTGGAGGTGGATCTT-3’ |
| Dof-type Zinc finger domain similar to zinc finger protein OBP4 (At5g60850) | EX930621 | Fwd5’-GCCGAGAAATTCCTGTACCA-3’  Rev 5’-GACCTGCGGTTCCTCTGTAA-3’ |
| Glycosyl hydrolase family protein (At2g03505) | EX931200 | Fwd 5’-AACACCCACAACCCCAGTAA-3’  Rev 5’-CACAGGCATAGTCCAGAGCA-3’ |
| Lipoxygenase (At1g72520) | EX930733 | Fwd 5’**-**TCTGGAGCTTCTCTCCAAGC-3’  Rev 5’-CTGCTCCAACGACAATGAGA-3’ |
| Phospholipase D/PLD Delta (At4g35790) | EX931456 | Fwd 5’-CTGGGAAGCTCTTTTGGATG-3’  Rev 5’-GAAGCACTCTCTGGCACTCC-3’ |
| Guanylate kinase (GK-2) (At3g57550) | EX931690 | Fwd 5’-CAGCTTTAGGCTCCCCTTTT-3’  Rev 5’-AACAGCATCTTTCCCCACAC-3’ |
| RNA binding protein 37 (At3g49390) | EX931655 | Fwd 5’-TCACTTTGTGGGGAGGTTTC-3’  Rev 5’-GGGGAATGCGAGGTCTAACT-3’ |
| Protein phosphatase 2C family protein / PP2C family protein (At5g66080) | EX931491 | Fwd 5’-GATGGGAAGCAGTTGTTGGT-3’  Rev 5’-TACACGAGGCACATCTCCTG-3’ |
| 40S Ribosomal protein RPS15E (At5g43640) | EX930206 | Fwd 5’-CCAACGAGGTTTGAAGAGGA-3’  Rev 5-CGAAGGTGGGTTCTAACTGG-3’ |
| ABC Transporter (At5g19410) | EX931067 | Fwd 5’-GGTCATGGTTCTGCCAAACT-3’  Rev 5’-GTCTCGGGAGTGTACCCAAA-3’ |
| Cytochrome p450 family protein (At5g44620) | EX931477 | Fwd 5’-GAGTCAATCTGGGGACCTGA-3’  Rev 5’-TCTGCAAATAGGCCAAATCC-3’ |
| Protein kinase, putative similar to protein kinase APK1A (At3g01300) | EX930573 | Fwd 5’-GCCAAGCTTTCTGACTTTGG-3’  Rev 5’-GTTTAGCCCATTCCACCAGA-3’ |
| Homeobox protein knotted-1 like 4 (KNAT4) (At5g11060) | EX930623 | Fwd 5’-CTCCCAGAGCGATCAGTTTC-3’  Rev 5’-TCCACCATAGGCTTCCAGAG-3’ |
| F-box family protein (At5g53200) | EX930398 | Fwd 5’-GCACAGGCCTGATCATGTAA-3’  Rev 5’-ATCCCCAGCAAATGCTACTG-3’ |
| MYB transcription factor (At5g59570) | EX931599 | Fwd 5’-CGAACACCAACACAAGTTGCC-3’  Rev 5’-TTGGGGGCTGACTAATTGA-3’ |
| Importin alpha subunit IMPA4 (At1g09270) | EX931076 | Fwd 5’-TTGGCTGGAAGATGAAGAGG-3’  Rev 5’-CAGATGGAACTTGTGCCTGA-3’ |
| Plasma membrane intrinsic protein 1C (PIP1C) / aquaporin PIP1.3 (PIP1.3) / transmembrane protein B (TMPB) (At1g01620) | EX930884 | Fwd 5’-CTTCCAGCAGAGCGAGTACC-3’  Rev 5’-GAGCCAGAAGAGGAACATGC-3’ |
| ATP dependent RNA helicase (At1g59760) | EX931761 | Fwd 5’-TCGGATTGGAAGAACAGGAC-3’  Rev 5’-CCTAGCAGCCTTTCCAGTTG-3’ |

| **REFERENCE (HOUSEKEEPING) GENES** | | |
| --- | --- | --- |
| **Description** | **GenBank Acc.** | **Primers** |
| Actin 2 | EX930365 | Fwd 5’**-**TGTGCTCAGTGGTGGGTCTAG-3’  Rev 5’-GTGCAACCACCTTGATCTT-3’ |
| 18S Ribosomal | NR_022795 | Fwd 5’-ATGATAACTCGACGGATCGC-3’  Rev 5’-CTTGGATGTGGTAGCCGTTT-3’ |
| Glyceraldehyde-3-phosphate dehydrogenase (GDH) | Q39769.1 | Fwd 5’AGGAGGAATCTGAGGGGAAA  Rev 5’-TCACCCGAGAACTGTAACCC-3’ |
| **GENES INVOLVED IN PHENYLPROPANOID BIOSYNTHESIS** | | |
| **Description** | **GenBank Acc.** | **Primers** |
| Phenylalanine ammonia-lyase | AY231176.1 | Fwd 5’-CGCGAGAAAGCTCCATAAAC-3’  Rev 5’-GAGAAGAGTTTGCCCATCCA-3’ |
| Chalcone synthase | EU391660.1 | Fwd 5’ATGTACCAGCAAGGCTGCTT-3’  Rev 5’-GTCACCGCCGTTATTTCACT-3’ |
| Flavanone 3-hydroxylase | AY742228.1 | Fwd 5’-ATACAAGAACGCGGACCATC-3’  Rev 5’-CACTCCTTCCAGGGGATACA-3’ |
| Flavonol synthase 1 | GQ994432.1 | Fwd 5’-CGTAAGGCCTGTTGAGGAAA-3’  Rev 5’-AGGGAGAGATGGCATGATTG-3’ |
| Anthocyanidin reductase | AY750963.1 | Fwd 5’-CTTCCTGACAAGCCAAAAGC-3’  Rev 5’-AGGGCAAGTTCAACGCTAGA-3’ |
| **ROS SCAVENGING GENES** | | |
| **Description** | **GenBank Acc.** | **Primers** |
| Ascorbate peroxidase | FJ555021.1 | Fwd 5’-CTGGTGTGGTTGCTGTTGAG-3’  Rev 5’-GTGGCATCTTCCCAAAGTGT-3’ |
| Peroxidase | FJ599670.1 | Fwd 5’-AAACTGTCTCCTGCGCTGAT-3’  Rev 5’-AAGGTCGGTTGTGTCCAGAG-3’ |
| Superoxide dismutase | EF633506.1 | Fwd 5’-TAGACCGAAGGCCGTAGAGA-3’  Rev 5’-GGCTCCAGCGCACTATAATC-3’ |
| Catalase | FJ555022.1 | Fwd 5’-CCCACCTGTGGACAGAAGTT-3’  Rev 5’-TATCCTCATCTGCCGGATTC-3’ |
| **GENES INVOLVED IN TERPENOID BIOSYNTHESIS** | | |
| **Description** | **GenBank Acc.** | **Primers** |
| Levopimaradiene synthase | AY574248.2 | Fwd 5’-CGGTCCAAATTTCTGCATCT-3’  Rev 5’-TCAATTCCTTCAGTGCGTTG-3’ |
| Farnesyl diphosphate synthase | AY389818.1 | Fwd 5’-TCATTCTATCTCCCGGTTGC-3’  Rev 5’-TGGATCACCAAAGCAATCAA-3’ |
| Geranylgeranyl diphosphate synthase | AY371321.1 | Fwd 5’-AGGGCTTGTAGCTGGACAGA-3’  Rev 5’-CACTCCTTCCAGGGGATACA-3’ |
